# Chemical, mineral composition, and sensory acceptability of cocoyam‐based recipes enriched with cowpea flour

**DOI:** 10.1002/fsn3.30

**Published:** 2013-03-06

**Authors:** Ibiyemi Olayiwola, Funmi Folaranmi, Abdul‐Rasaq A. Adebowale, Onabanjo Oluseye, Sanni Ajoke, Afolabi Wasiu

**Affiliations:** ^1^ Department of Nutrition and Dietetics Federal University of Agriculture Abeokuta Ogun Nigeria; ^2^ Department of Food Science and Technology Federal University of Agriculture Abeokuta Ogun Nigeria

**Keywords:** Cocoyam‐based recipes, cowpea flour, development, nutritional evaluation, protein quality, sensory evaluation

## Abstract

The study was conducted to improve cocoyam‐based recipes (steamed cocoyam paste [*ebiripo*], ikokore, and fried cocoyam balls [*ojojo*]) using different blends of cocoyam and cowpea flours (100:0, 80:20, 70:30, 60:40, and 50:50). The proximate composition, mineral composition, and sensory qualities of the recipes were determined using standard analytical procedures. The recipes had significantly (*P *<* *0.05) higher contents of protein, fat, crude fiber, iron, zinc, sodium, and phosphorus compared with the control recipe (100% cocoyam flour). The protein content was highest in all recipes containing 50:50 cocoyam: cowpea flour (10.79%, 10.56%, 10.36% for *ojojo, ikokore,* and *ebiripo*, respectively). However, *ikokore* had higher iron (2.5 mg), phosphorus (92.5 mg), and zinc (1.92 mg) contents than *ebiripo* and *ojojo*. While the 80:20 recipe for *ebiripo* had significantly (*P *<* *0.05) higher flavor and overall acceptability scores compared with other recipes. In conclusion, enrichment of cocoyam‐based recipes with cowpea flour improved the proximate composition, mineral composition, and sensory acceptability of the foods.

## Practical Applications

The dietetic value of cocoyam–cowpea blends is enormous in many hospitals, especially in Nigeria. Dieticians in Africa and other cocoyam‐consuming countries (especially developing countries) will find the combination of cocoyam and cowpea highly valuable in the treatment of malnutrition and other chronic diseases that are caused by protein deficiency.

## Introduction

Cocoyam (*Colocasia esculenta*) is a well‐known food plant that has a long history of cultivation. Its corms are important sources of starch. Cultivars of two species *Colocasia esculenta* (taro) and *Xanthosoma sagittifolium* (tannia) are generally grown for food. It is consumed in homes, especially during periods preceding the yam harvest, which underscores its importance as a possible substitute for the crop (Ajijola et al. 2003). It is used essentially the same way as yam, although it is not as highly valued. Cocoyam is a perfect complementary element for all sorts of meals, as it offers vitamins and soluble fibers (Niba 2003; Ndabikunze et al. 2011).

Cocoyam ranks third in importance after cassava and yam among the root and tuber crops that are cultivated and consumed in rural areas by the elderly in Nigeria. The crop is no longer favored in urban homes due to poor information about its nutritive values. The widespread ignorance of the nutritive value and diversities of foods from cocoyam constitutes a major impediment to its general acceptability and wider cultivation. Therefore, further efforts are needed to popularize its production among farmers and enhance its food use among consumers (Okoye et al. 2008; Adejumo and Bamidele 2012).

The main nutrient supplied by cocoyam, as with other roots and tubers, is dietary energy provided by its carbohydrate content. Its protein content is low (1–2%), and as in almost all root crop proteins, sulfur‐containing amino acids are limiting. By contrast, cowpea protein is of higher value and can complement the deficiencies of cocoyam. Cowpea is popularly referred to as “beans” in Nigeria and it belongs to the group known as legumes. It is nutritious and provides protein, vitamins, and minerals. Cowpea grain contains about 25% protein (Singh and Singh 1992), making it extremely valuable for people who cannot afford animal protein foods such as meat and fish (Bradbury and Holloway 1998). The use of cowpea seed as a vegetable provides an inexpensive source of protein in the diet. The plant can be used at all stages of growth as a vegetable crop. The combination of cocoyam–cowpea flour could be used in the industrial production of baked foods, noodles, and weaning foods (Ugwu 2009; Rita and Sophia 2010; Agunbiade et al. 2011).

It has been documented in literatures that blends of cocoyam and cowpea flour will improve the protein content of cocoyam flour (Davidson et al. 1979; Singh and Singh 1992). Furthermore, the dietetic value of cocoyam–cowpea blends is enormous in many hospitals, especially in Nigeria. Dieticians in Africa will find the combination of cocoyam and cowpea highly valuable in the treatment of malnutrition and other chronic diseases that depend on vegetable proteins. Therefore, the objective of this study was to develop cocoyam flour‐based recipes enriched with cowpea and determine the proximate and mineral composition as well as sensory acceptability of the developed recipes.

## Materials and Methods

### Raw materials

The cocoyam cormels and cowpea grains used in this study were purchased from Osiele and Kuto markets, Abeokuta, Ogun State, Nigeria. The food items (steamed cowpea paste [*ebiripo*]*,* fried cowpea balls [*ojojo*], and cocoyam pudding [*ikokore*]) were prepared at the Food Preparation Laboratory of Nutrition and Dietetics Department, Federal University of Agriculture, Abeokuta, Nigeria.

### Preparation of cocoyam flour

The cocoyam cormels were thoroughly washed with water, peeled, and grated into wet mash using a manual kitchen grater. They were oven dried at a temperature of 65°C for 48 h. After drying, they were milled finely and stored in air‐tight polyethylene bags and stored at 4°C until use.

### Preparation of cowpea flour

The cowpea was processed according to the method of Nnanna and Phillips (1990). Cowpea seeds were cleaned, soaked, and then dehulled. The detached hulls were decanted from the beans. The dehulled beans were then dried at a temperature of 65°C in a hot air oven for 48 h, milled into flour, packed in an air‐tight container, and stored.

### Preparation of fried cocoyam balls (*ojojo*)

Cocoyam flour alone was used to prepare the control *ojojo*. Varying composites of cocoyam and cowpea flour in the ratio of 50:50, 60:40, 70:30, and 80:20 were used to prepare *ojojo*. Water was added to form a paste of soft consistency. These pastes were mixed with the appropriate ingredients (HETAN 1999) for frying *ojojo* (pepper, salt). A deep spoon was used to drop the mixture into the hot oil, thereby forming ball shapes. The mixture was then deep fried in hot oil (about 170°C for 5 min) until light brown. The standardized recipe is shown in Table 1.

**Table 1:** Standardized recipe for fried cocoyam ball (*ojojo*)

Ingredients	Weight
Cocoyam flour and cowpea flour	900 g
Onion	40 g
Pepper	50 g
Water to mix	600 mL
Salt	1 g%
Vegetable oil	For frying

### Preparation of cocoyam pudding (*ikokore*)

The composite flour (50:50, 60:40, 70:30, and 80:20 cocoyam: cowpea) was weighed into a bowl and mixed with a small quantity of warm water to obtain a soft and smooth consistency. Salt, ground pepper, and onion were added to taste before thoroughly mixing. A small quantity of palm oil was poured into the pot (to prevent it from burning), cent leaves were washed and used as base, and then smoked fish was put on top of the base. The cocoyam–cowpea paste was added in small even sizes on the smoked fish. Another small quantity of warm water was added to improve consistency and allowed to boil for 10 min. The dried fish and remaining oil were added, allowed to cook for 20 min, and then it was stirred and ready to be served. The standardized recipe is presented in Table 2.

**Table 2:** Standardized recipe for cocoyam pudding (*ikokore*)

Ingredients	Weight
Cocoyam flour and cowpea flour	1000 g
Onion	40 g
Pepper	50 g
Water to mix	500 mL
Salt	To taste
Smoked fish	200 g
Palm oil	200 mL

### Preparation of steamed cocoyam paste (*ebiripo*)

The ground ingredients (pepper, onion, curry, thyme) were added to the cocoyam–cowpea composite flour. Palm oil with warm water was added to make a slurry of soft and smooth consistency. Salt was added to taste. The mixture was wrapped in local leaves and then steam cooked for about 30 min. The standardized recipe for steamed cocoyam paste preparation is shown in Table 3.

**Table 3:** Standardized recipe for steamed cocoyam paste (*ebiripo*)

Ingredients	Weight
Cocoyam flour and cowpea flour	1000 g
Pepper	50 g
Palm oil	200 mL
Salt	1 g%
Ground shrimps	30 g
Water	600 mL
Leaves	For wrapping

### Analyses

#### Determination of proximate composition

The moisture, fat, protein, ash, and crude fiber contents were determined using Association of Official Analytical Chemists (AOAC 2005) methods. The carbohydrate content was determined by the difference between 100 and the total sum of the percentage of fat, moisture, ash, crude fiber, and protein content.

#### Determination of mineral composition

The mineral content in each sample was analyzed using atomic absorption spectrophotometer (AAS) fitted with a hollow cathode lamp and a fuel‐rich flame (air‐acetylene) following the AOAC (2005) procedure.

#### Sensory evaluation

The sensory evaluation of the products was performed using a 9‐point hedonic scale ranking 0–8 (0 = extremely dislike and 8 = extremely like). The panelists assessed the product for flavor, fluffiness, color, taste, and overall acceptability (Iwe 2002 *;* Ukpabi 2003).

### Statistical analysis

Data were analyzed using analysis of variance (ANOVA) and Duncan multiple range test to test significant differences between means (*P *<* *0.05). Statistical analysis was done using Statistical Package for Social Science (SPSS) version 16.0 (IBM SPSS, Inc., Chicago, IL).

## Results

### Proximate composition

The results of the proximate composition of the products from cocoyam and cowpea flour composites are presented in Tables 4, 5 and 6.

**Table 4:** Proximate composition of fried cocoyam balls (*ojojo)*

Cocoyam–cowpea flour	Moisture content (%)	Protein (%)	Fat (%)	Ash (%)	Crude fiber (%)	Carbohydrate (%)
Control	54.06^b^ ± 0.01	2.41^e^ ± 0.06	12.9^d^ ± 0.02	2.1^d^ ± 0.00	2.35^d^ ± 0.01	28.51^a^ ± 0.06
80:20	53.58^c^ ± 0.24	5.66^d^ ± 0.04	12.98^d^ ± 0.08	2.53^c^ ± 0.07	2.74^c^ ± 0.03	25.23^c^ ± 0.16
70:30	51.36^d^ ± 0.11	7.35^c^ ± 0.01	13.38^c^ ± 0.01	2.61^bc^ ± 0.01	2.75^bc^ ± 0.03	26.28^b^ ± 0.14
60:40	56.08^a^ ± 0.01	9.09^b^ ± 0.12	13.56^b^ ± 0.06.	2.71^ab^ ± 0.01	2.84^ab^ ± 0.02	18.54^d^ ± 0.16
50:50	46.23^e^ ± 0.14	10.79^a^ ± 0.05	14.75^a^ ± 0.05	2.80^a^ ± 0.02	2.91^a^ ± 0.01	25.42^c^ ± 0.14

±Standard deviation of three replicates.

Mean values along the same column with different superscripts are significantly different (*P* ≤ 0.05).

**Table 5:** Proximate composition of cocoyam pudding (*ikokore)*

Cocoyam–cowpea flour	Moisture content (%)	Protein (%)	Fat (%)	Ash (%)	Crude fiber (%)	Carbohydrate (%)
Control	54.86^d^ ± 0.13	3.55^e^ ± 0.07	9.19^d^ ± 0.09	2.3^c^ ± 0.11	2.54^e^ ± 0.02	30.09^a^ ± 0.11
80:20	59.47^c^ ± 0.25	5.90^d^ ± 0.13	9.41^c^ ± 0.01	2.78^b^ ± 0.03	2.81^d^ ± 0.01	22.42^b^ ± 0.29
70:30	64.28^a^ ± 0.26	7.71^c^ ± 0.08	9.73^b^ ± 0.04	2.89^b^ ± 0.02	2.92^c^ ± 0.01	15.38^c^ ± 0.34
60:40	62.36^b^ ± 0.09	9.10^b^ ± 0.07	9.95^a^ ± 0.08	2.95^ab^ ± 0.01	3.01^b^ ± 0.01	15.63^c^ ± 0.23
50:50	63.39^a^ ± 0.39	10.56^a^ ± 0.13	10.06^a^ ± 0.01	3.20^a^ ± 0.15	3.08^a^ ± 0.01	12.77^d^ ± 0.52

±Standard deviation of three replicates.

Mean values along the same column with different superscripts are significantly different (*P* ≤ 0.05).

**Table 6:** Proximate composition of steamed cocoyam paste (*ebiripo)*

Cocoyam–cowpea flour	Moisture content (%)	Protein (%)	Fat (%)	Ash (%)	Crude fiber (%)	Carbohydrate (%)
Control	58.38^a^ ± 0.07	2.83^e^ ± 0.03	11.85^e^ ± 0.06	2.16^d^ ± 0.06	2.32^e^ ± 0.00	24.77^a^ ± 0.11
80:20	55.42^b^ ± 0.15	5.66^d^ ± 0.07	12.01^d^ ± 0.02	2.63^c^ ± 0.06	2.62^d^ ± 0.00	24.26^ab^ ± 0.13
70:30	53.94^c^ ± 0.29	7.29^c^ ± 0.01	12.28^c^ ± 0.00	2.71^bc^ ± 0.04	2.73^c^ ± 0.01	23.76^b^ ± 0.31
60:40	51.28^d^ ± 0.26	8.92^b^ ± 0.02	12.44^b^ ± 0.03	2.81^ab^ ± 0.01	2.77^b^ ± 0.01	24.52^a^ ± 0.24
50:50	49.51^e^ ± 0.23	10.36^a^ ± 0.17	12.71^a^ ± 0.06	2.88^a^ ± 0.02	2.88^a^ ± 0.00	24.51^a^ ± 0.09

±Standard deviation of three replicates.

Mean values along the same column with different superscripts are significantly different (*P* ≤ 0.05).

The moisture content values for *ojojo* ranged from 46.23% to 54.06%. The mean protein value was 2.4% for 100% cocoyam, and this increased as the percentage of cowpea flour increased. The blended ratio 50:50 cocoyam: cowpea flour had the highest protein content. The fat content also increased with increasing cowpea flour; *ojojo* with a 50:50 flour blend had the highest fat content (14.75%). The mean ash content value for *ojojo* was 2.1%. The crude fiber contents were 2.35% for 100% cocoyam, 2.74% for 80:20, 2.75% for 70:30, 2.84% for 60:40, and 2.91% for 50:50. The carbohydrate values of *ojojo* were 28.51% for 100% cocoyam, 25.23% for 80:20, 26.28% for 70:30, 18.54% for 60:40, and 25.42% for 50:50.

The mean moisture contents of *ikokore* were 54.86%, 59.47%, 64.28%, 62.36%, and 63.39% for 100% cocoyam, 80:20, 70:30, 60:40, and 50:50 blends, respectively. The *ikokore* from 50:50 composite flour recorded the highest protein (10.56%), fat (10.06%), and ash (3.08%) contents. The carbohydrate content was highest for 100% cocoyam flour (30.09%), and decreased with increasing addition of cowpea flour.

The mean moisture content of *ebiripo* was highest in 100% cocoyam (58.38%). The *ebiripo* from 50:50 composite had the highest values for protein (10.36%), fat (12.71%), ash (2.88%), and crude fiber (2.88%), whereas carbohydrate was highest in 100% cocoyam sample. *Ebiripo* had the highest moisture content followed by *ikokore* and *ojojo*, respectively.

The protein content of cocoyam was 1.5%, which is low compared with the protein content of 23.0% in cowpea. The protein content of the recipes developed in this study increased as the percentage of cowpea flour increased. The control sample of *ojojo* had the lowest percentage of protein (2.41%) when compared with the control samples of *ikokore* and *ebiripo*, which had protein contents of 3.5% and 2.83%, respectively. Samples of the recipes developed with 50% cowpea flour substitution were significantly higher (*P *<* *0.05) in protein (10.79%, 10.56%, and 10.36% for *ojojo, ikokore*, and *ebiripo*, respectively) than the samples with >40% cowpea flour substitution. The percentage fat content of all the developed recipes differ significantly (*P *<* *0.05), except the cowpea flour substitution of 40% and 50% for *ikokore*. The fat content increased with the increase in cowpea flour. The fiber content of the products ranged from 2.32% to 3.08%. The 50% cowpea flour substitution of *ikokore* was significantly higher in crude fiber (3.08%) at *P *<* *0.05, whereas the *ebiripo* control sample had the lowest crude fiber percentage (2.32%). The percentage ash contents were greatest in all the 50% cowpea flour‐substituted recipes (i.e., *ojojo*,* ikokore*, and *ebiripo*), although *ikokore* had the highest percentage ash content of 3.20% when compared with *ojojo* and *ebiripo* with 2.8% and 2.88%, respectively. The carbohydrate content decreased (from 30.09% to 12.77%) with an increase in the percentage (from 20% to 50%) of cowpea flour. There was no significant difference (*P *>* *0.05) in the carbohydrate contents of the *ebiripo* samples. However, the percentage carbohydrate content of *ojojo* and *ikokore* samples differs significantly above 20% addition of cowpea flour (*P < *0.05).

### Mineral composition

The results of mineral composition of the recipes developed are shown in Tables 7, 8 and 9.

**Table 7:** Mineral composition (mg/100 g) of fried cocoyam balls (*ojojo)*

Cocoyam–cowpea flour	Calcium	Iron	Potassium	Phosphorus	Sodium	Zinc
Control	51.7^a^ ± 0.11	0.700^e^ ± 0.2	1113.8^a^ ± 2.63	59.83^e^ ± 0.03	277.6^e^ ± 0.10	0.40^e^ ± 0.00
80:20	45.36^b^ ± 0.03	1.0^d^ ± 0.00	1039.2^b^ ± 0.05	72.13^d^ ± 0.08	484.8^d^ ± 0.01	0.78^d^ ± 0.00
70:30	42.1^c^ ± 0.03	1.10^c^ ± 0.00	987.8^c^ ± 0.05	78.30^c^ ± 0.05	513.4^c^ ± 0.02	0.95^c^ ± 0.00
60:40	39.8^d^ ± 0.05	1.19^b^ ± 0.00	834.4^d^ ± 0.05	83.9^b^ ± 0.00	801^b^ ± 0.19	1.2^a^ ± 0.00
50:50	35.5^e^ ± 0.00	1.3^a^ ± 0.00	795.5^e^ ± 0.00	91.5^a^ ± 0.57	1158.8^a^ ± 0.01	1.17^b^ ± 0.00

±Standard deviation of three replicates.

Mean values along the same column with different superscripts are significantly different (*P* ≤ 0.05).

**Table 8:** Mineral composition (mg/100 g) of cocoyam pudding (*ikokore)*

Cocoyam–cowpea flour	Calcium	Iron	Potassium	Phosphorus	Sodium	Zinc
Control	54.43^a^ ± 0.23	0.91^e^ ± 0.2	1125.5^a^ ± 0.26	63.4^e^ ± 0.11	434^e^ ± 0.09	0.44^e^ ± 0.00
80:20	45.56^b^ ± 0.06	1.2^d^ ± 0.05	1065.5^b^ ± 0.27	73.6^d^ ± 0.11	681.8^d^ ± 0.01	0.95^d^ ± 0.00
70:30	39.81^c^ ± 0.01	1.70^c^ ± 0.00	1015.3^c^ ± 0.20	79.16^c^ ± 0.06	864.1^c^ ± 0.04	1.36^c^ ± 0.00
60:40	34.71^d^ ± 0.00	2.15^b^ ± 0.02	942.08^d^ ± 0.01	85.8^b^ ± 0.00	1172^b^ ± 0.08	1.74^b^ ± 0.00
50:50	32.85^e^ ± 0.01	2.50^a^ ± 0.00	897.33^e^ ± 0.08	92.5^a^ ± 0.00	1407.97^a^ ± 0.01	1.92^a^ ± 0.00

±Standard deviation of three replicates.

Mean values along the same column with different superscripts are significantly different (*P* ≤ 0.05).

**Table 9:** Mineral composition (mg/100 g) of steamed cocoyam paste (*ebiripo)*

Cocoyam–cowpea flour	Calcium	Iron	Potassium	Phosphorus	Sodium	Zinc
Control	56.3^a^ ± 0.11	0.77^e^ ± 0.01	1045.3^a^ ± 0.96	59.83^e^ ± 0.44	260^e^ ± 0.02	0.37^c^ ± 0.00
80:20	49.4^b^ ± 0.05	1.00^d^ ± 0.00	998.7^b^ ± 0.11	66.6^d^ ± 0.11	525^d^ ± 0.01	0.91^b^ ± 0.00
70:30	46.06^c^ ± 0.03	1.13^c^ ± 0.00	938.8^c^ ± 0.00	72.80^c^ ± 0.00	863^c^ ± 0.01	1.31^a^ ± 0.26
60:40	42.8^d^ ± 0.05	1.2^b^ ± 0.00	890.7^d^ ± 0.11	79.83^b^ ± 0.06	1118^b^ ± 0.01	1.2^ab^ ± 0.00
50:50	39.53^e^ ± 0.8	1.3^a^ ± 0.00	810.5^e^ ± 1.15	85.2^a^ ± 0.11	1273^a^ ± 0.01	1.55^a^ ± 0.00

±Standard deviation of three replicates.

Mean values along the same column with different superscripts are significantly different (*P* ≤ 0.05).

For 0–50% cowpea flour inclusion, *ikokore* had the highest value of iron, ranging from 0.91 to 2.5 mg/100 g, compared with *ojojo* with 0.70–1.30 mg/100 g and *ebiripo* with 0.77–1.30 mg/100 g. *Ikokore* had the highest value of phosphorous, ranging from 63.40 to 92.50 mg/100 g, compared with *ojojo* with values of 59.83–91.50 mg/100 g and *ebiripo* with values of 59.83–85.2 mg/100 g. *Ikokore* recorded the highest value of zinc, ranging from 0.44 to 1.92 mg/100 g, whereas *ojojo* had values of 0.40–1.17 mg/100 g and *ebiripo* had values of 0.37–1.55 mg/100 g.

It was observed from the table of results that the values of calcium and potassium in all the control recipes (100% cocoyam flour) of *ojojo, ikokore*, and *ebiripo* decreased with increasing cowpea flour inclusion. The calcium content of *ojojo* ranged from 51.7 to 35.50 mg/100 g, while *ebiripo* followed with values of 56.30–39.53 mg/100 g, and *ikokore* was the lowest with values of 54.43–32.85 mg/100 g. For potassium, *ikokore* had the least decrease of 1125.50–897.33 mg/100 g, followed by *ebiripo* with values of 1045.30–810.50 mg/100 g, and *ojojo* had the lowest value of 1113.80–795.50 mg/100 g.

### Sensory evaluation

The results of the sensory evaluation of the cocoyam–cowpea composite flour recipes are shown in Tables 10, 11 and 12. For *ojojo*, the control sample had the most acceptable color with a mean value of 6.44, whereas samples with 20% and 50% cowpea flour substitution had the least acceptable color with a mean value of 5.96. Also, samples of *ojojo* with 40% cowpea flour substitution had the most acceptable flavor with a mean value of 6.28, while the sample with the least acceptable flavor was 30% cowpea flour substitution with a mean value of 5.80. The 20% cowpea flour substitution had the most acceptable fluffiness with a mean value of 6.32, whereas the sample with 50% cowpea flour substitution had the least acceptable fluffiness with a mean value of 5.68. Lastly, the sample with 40% cowpea flour substitution had the highest taste acceptability with a mean value of 6.48, while the sample with 50% cowpea flour substitution had the least with a mean value of 5.72. In summary, samples of *ojojo* with 40% cowpea flour substitution had the highest mean value of overall acceptability of 6.64, whereas samples with 20% and 50% had the least overall acceptability with a mean value of 5.80.

**Table 10:** Effect of cowpea flour on the sensory characteristics of fried cocoyam paste (*ojojo*)

Cocoyam–cowpea flour	Color	Flavor	Fluffiness	Taste	Overall acceptability
Control	6.44^ab^ ± 0.18	6.20^a^ ± 0.16	6.12^ab^ ± 0.13	6.12^ab^ ± 0.23	6.16^ab^ ± 0.16
80:20	5.96^b^ ± 0.19	6.12^a^ ± 0.17	6.32^a^ ± 0.21	5.92^ab^ ± 0.14	5.80^b^ ± 0.14
70:30	6.08^b^ ± 0.18	5.80^a^ ± 0.19	5.96^ab^ ± 0.16	6.08^ab^ ± 0.20	5.88^b^ ± 0.19
60:40	6.96^a^ ± 0.19	6.28^a^ ± 0.14	6.12^ab^ ± 0.12	6.48^a^ ± 0.25	6.64^a^ ± 0.22
50:50	5.96^b^ ± 0.19	5.92^a^ ± 0.16	5.68^b^ ± 0.21	5.72^b^ ± 0.18	5.80^b^ ± 0.18

±Standard deviation of 30 panelist rating.

Mean values along the same column with different superscripts are significantly different (*P* ≤ 0.05).

**Table 11:** Effect of cowpea flour on the sensory characteristics of cocoyam pudding (*ikokore*)

Cocoyam–cowpea flour	Color	Flavor	Fluffiness	Taste	Overall acceptability
Control	5.32^c^ ± 0.17	5.48^c^ ± 0.15	5.56^b^ ± 0.19	5.36^c^ ± 0.22	5.20^c^ ± 0.22
80:20	6.00^b^ ± 0.24	5.84^bc^ ± 0.19	6.32^a^ ± 0.21	6.12^b^ ± 0.19	6.20^ab^ ± 0.20
70:30	6.28^b^ ± 0.18	6.12^ab^ ± 0.17	6.44^a^ ± 0.17	6.17^ab^ ± 0.21	6.52^a^ ± 0.17
60:40	6.92^a^ ± 0.20S	6.56^a^ ± 0.16	6.56^a^ ± 0.22	6.68^a^ ± 0.15	6.52^a^ ± 0.18
50:50	5.88^b^ ± 0.16	6.00^b^ ± 0.18	6.04^ab^ ± 0.17	6.04^b^ ± 0.17	5.92^b^ ± 0.17

±Standard deviation of 30 panelist rating.

Mean values along the same column with different superscripts are significantly different (*P* ≤ 0.05).

**Table 12:** Effect of cowpea flour on the sensory characteristics of steamed cocoyam paste (*ebiripo*)

Cocoyam–cowpea flour	Color	Flavor	Fluffiness	Taste	Overall acceptability
Control	7.48^a^ ± 0.14	7.24^a^ ± 0.14	7.48^a^ ± 0.13	7.88^a^ ± 0.06	7.28^a^ ± 0.11
80:20	7.00^b^ ± 0.14	7.24^a^ ± 0.14	7.04^b^ ± 0.12	7.32^b^ ± 0.15	7.52^a^ ± 0.12
70:30	5.96^c^ ± 0.16	5.60^b^ ± 0.17	5.76^c^ ± 0.16	6.16^c^ ± 0.17	6.48^b^ ± 0.14
60:40	5.64^c^ ± 0.14	5.52^b^ ± 0.16	6.12^c^ ± 0.13	6.32^c^ ± 0.19	5.84^c^ ± 0.14
50:50	5.20^d^ ± 0.13	5.52^b^ ± 0.12	5.04^d^ ± 0.19	6.40^c^ ± 0.22	6.04^c^ ± 0.21

±Standard deviation of 30 panelist rating.

Mean values along the same column with different superscripts are significantly different (*P* ≤ 0.05).

For *ikokore*, the sample with 40% cowpea flour substitution had the most acceptable color, flavor, fluffiness, and taste with mean values of 6.92, 6.56, 6.56, and 6.68, respectively. The control sample (100% cocoyam) had the least acceptable color, flavor, fluffiness, and taste with mean values of 5.32, 5.48, 5.56, and 5.36, respectively. In summary, samples of *ikokore* with 40% and 30% cowpea flour substitution had the highest mean value of overall acceptability of 6.52, whereas the control sample (100% cocoyam) had the least mean value of overall acceptability of 5.20.

For *ebiripo,* the control sample had the highest acceptability for color, flavor, fluffiness, and taste, with mean values of 7.48, 7.24, 7.48, and 7.88, respectively. Samples with 50% cowpea flour substitution had the least acceptability for color, flavor, and fluffiness with mean values of 5.20, 5.52, and 5.04, respectively. Samples with 30% cowpea flour substitution had the least acceptability for taste with a mean value of 6.16. In summary, the samples with 20% cowpea flour substitution had the most overall acceptability with a mean value of 7.52, whereas samples with 40% cowpea flour substitution had the least overall acceptability with a mean value of 5.84.

## Discussion

The inclusion of cowpea flour significantly increased the protein for all samples (*ojojo*,* ikokore*, and *ebiripo*). This expected increase in the protein of the blends is the basis for the enrichment such that the final product will have higher protein quantity and quality. Because tuber crops including cocoyam are low in sulfur amino acids (Fashakin et al. 1986; WHO/UNICEF 1988), the proteins in cowpea flour will complement those of cocoyam and thus improve the nutritional quality of traditional recipes. There is a general increase in the fat and crude fiber content with increasing amount of cowpea flour to cocoyam. The importance of fiber in the diet of man cannot be overemphasized to improve laxation, reduce diverticular disease and obesity, and in its dietetic value for diabetes.

Ash is a nonorganic compound reflecting the mineral content of food. Nutritionally, ash aids in the metabolism of other organic compounds such as carbohydrate and fat (McWilliams 1978). The percentage ash, which is an indicator of the mineral content of the product, increased with an increase in the percentage of cowpea in all the developed products. Iron, phosphorus, and zinc contents were highest in 50% cowpea flour‐substituted samples of *ikokore*,* ojojo*, and *ebiripo*. By contrast, calcium and potassium contents decreased with increasing percentage of cowpea flour substitution in all the samples.

The control sample (100% cocoyam) had the most acceptable color for all the products. However, the sample with 40% cowpea flour substitution was most acceptable in terms of flavor, taste, and overall acceptability. This result also could be attributed to the bean flavor of cowpea. The sample with 20% cowpea flour substitution had the most acceptable texture (fluffiness).

## Conclusions

This study has shown that significant improvement in the proximate composition, mineral composition, and sensory acceptability is achievable through enrichment of cocoyam flour‐based recipes with cowpea flour.

## Conflict of Interest

None declared.
